# Gammaherpesviral Gene Expression and Virion Composition Are Broadly Controlled by Accelerated mRNA Degradation

**DOI:** 10.1371/journal.ppat.1003882

**Published:** 2014-01-16

**Authors:** Emma Abernathy, Karen Clyde, Rukhsana Yeasmin, Laurie T. Krug, Al Burlingame, Laurent Coscoy, Britt Glaunsinger

**Affiliations:** 1 Department of Plant and Microbial Biology, University of California at Berkeley, Berkeley, California, United States of America; 2 Department of Computer Science, Stony Brook University, Stony Brook, New York, United States of America; 3 Department of Molecular Genetics and Microbiology, Stony Brook University, Stony Brook, New York, United States of America; 4 Department of Pharmaceutical Chemistry, University of California at San Francisco, San Francisco, United States of America; 5 Department of Cell and Molecular Biology, University of California at Berkeley, Berkeley, California, United States of America; University of Colorado Denver School of Medicine, United States of America

## Abstract

Lytic gammaherpesvirus infection restricts host gene expression by promoting widespread degradation of cytoplasmic mRNA through the activity of the viral endonuclease SOX. Though generally assumed to be selective for cellular transcripts, the extent to which SOX impacts viral mRNA stability has remained unknown. We addressed this issue using the model murine gammaherpesvirus MHV68 and, unexpectedly, found that all stages of viral gene expression are controlled through mRNA degradation. Using both comprehensive RNA expression profiling and half-life studies we reveal that the levels of the majority of viral mRNAs but not noncoding RNAs are tempered by MHV68 SOX (muSOX) activity. The targeting of viral mRNA by muSOX is functionally significant, as it impacts intracellular viral protein abundance and progeny virion composition. In the absence of muSOX-imposed gene expression control the viral particles display increased cell surface binding and entry as well as enhanced immediate early gene expression. These phenotypes culminate in a viral replication defect in multiple cell types as well as *in vivo*, highlighting the importance of maintaining the appropriate balance of viral RNA during gammaherpesviral infection. This is the first example of a virus that fails to broadly discriminate between cellular and viral transcripts during host shutoff and instead uses the targeting of viral messages to fine-tune overall gene expression.

## Author Summary

Many viruses restrict host gene expression during infection, presumably to provide a competitive expression advantage to viral transcripts. Not surprisingly, viruses that induce this ‘host shutoff’ phenotype therefore generally possess mechanisms to selectively spare viral genes. Gammaherpesviruses promote host shutoff by inducing widespread mRNA degradation, a process initiated by the viral SOX nuclease. However, the effect of SOX on viral mRNA during infection was unknown. Here, we reveal that during infection with the murine gammaherpesvirus MHV68, the majority of viral transcripts of all kinetic classes are broadly down regulated through the activity of the MHV68 SOX protein (muSOX). We further demonstrate that in the absence of muSOX-induced control of viral mRNA abundance, viral protein levels increase, thereby affecting the composition of progeny viral particles. Altered virion composition directly impacts early events such as entry and induction of lytic gene expression in subsequent rounds of replication. Furthermore, decreasing both virus and host gene expression via global mRNA degradation is critical for viral replication in a cell type specific manner both *in vitro* and *in vivo*. This is the first example of a eukaryotic virus whose host shutoff mechanism similarly tempers viral gene expression, and highlights the degree to which gammaherpesviral gene expression must be fine tuned to ensure replicative success.

## Introduction

Viruses use a variety of mechanisms to dampen host gene expression, including inhibiting cap-dependent translation, transcription, splicing, and promoting host mRNA degradation. The presumed viral benefits of this ‘host shutoff’ phenotype include reduced competition for gene expression machinery and resources, as well as impaired immune responses through decreasing host factors involved in sensing infection. The importance of this phenotype *in vivo* has been directly confirmed for both alpha- and gammaherpesviruses, where host shutoff mutants exhibit defects in immune evasion (in the case of the herpes simplex viruses HSV-1 and HSV-2), viral trafficking, and latency establishment [1]–[3].

In all cases studied to date, viral transcripts largely escape the effects of host shutoff, thus affording them a competitive expression advantage. For example, poliovirus inhibits cap-dependent translation by cleaving eIF4G, thus enhancing translation of viral mRNAs that contain an internal ribosome entry site (IRES) but not a 5′ cap [4]–[6]. One mechanism of HSV-induced host shutoff involves altering phosphorylation of SR proteins to inhibit spliceosome assembly and block the biogenesis of nascent host mRNAs, the vast majority of which contain introns [7]. In contrast, HSV mRNAs are largely unspliced, enabling them to circumvent this block and, furthermore, are preferentially exported to the cytoplasm by the ICP27 protein [8], [9]. HSV-1 also promotes endonucleolytic cleavage of host mRNAs through its virally encoded ribonuclease vhs, which is packaged into viral particles and can thus impact host gene expression immediately after viral entry [2], [10]. Although HSV-1 mRNAs can be degraded by vhs in the absence of infection, recent data suggest that vhs is regulated by other viral factors in a manner that restricts its activity against viral RNA, particularly during delayed early and late gene expression [11], [12]. SARS coronavirus also causes host shutoff by promoting endonucleolytic cleavage of cellular mRNAs, but its viral mRNAs bear a protective 5′ leader sequence that prevents their cleavage [13].

Similar to alphaherpesviruses and SARS coronavirus, gammaherpesviruses promote host shutoff by inducing widespread cellular mRNA degradation [14], [15]. This viral subfamily includes the oncogenic human pathogens Kaposi's sarcoma-associated herpesvirus (KSHV) and Epstein-Barr virus (EBV), as well as the murine herpesvirus MHV68, a widely used model for understanding gammaherpesviral replication and pathogenesis *in vivo*. Gammaherpesviruses encode a viral nuclease termed SOX in KSHV that endonucleolytically cleaves cytoplasmic mRNAs during lytic infection, leading to their degradation by the host exoribonuclease Xrn1 [16]. SOX targets the majority of host mRNAs, and the ensuing depletion of cytoplasmic poly(A) mRNA causes nuclear relocalization of cytoplasmic poly(A) binding protein (PABPC), aberrant polyadenylation of RNAs in the nucleus, and an mRNA export defect [17]–[19]. These downstream phenotypes effectively magnify the gene expression block. Global mRNA destruction is mechanistically conserved in the SOX homologs in EBV (termed BGLF5) and MHV68 (termed muSOX), as are the PABPC relocalization and RNA processing and export sequelae [15], [20], [21]. This activity likely contributes to viral immune evasion, as it has been shown that the RNA degradation function of EBV BGLF5 impairs CD8+ T cell recognition in cultured cells [22]. Additionally, MHV68 bearing a muSOX mutant specifically defective for mRNA cleavage exhibits *in vivo* defects in viral trafficking from the mouse lung to distal sites, as well as a marked reduction in viral loads during peak latency establishment [1]. Thus, widespread mRNA degradation during lytic replication of gammaherpesviruses contributes measurably to the *in vivo* viral lifecycle, as well as to its interactions with the host immune system.

The prevailing assumption has been that host mRNA degradation is the driver of these phenotypes and, similar to other viruses studied to date, that viral transcripts must possess some mechanism to escape degradation. However, the susceptibility of viral transcripts to SOX-induced cleavage during infection has yet to be directly addressed, although they do not possess any common sequences that might aid in their escape. SOX and muSOX are expressed with early gene kinetics beginning at 8–10 hours post infection (hpi) and continuing through the end of the viral lifecycle [15]. It has therefore been presumed that, at a minimum, viral gene expression prior to the onset of host shutoff would be unaffected by SOX or muSOX activity. Here, we challenge both of these assumptions by showing that, unexpectedly, all stages of viral gene expression are strongly influenced by muSOX-induced RNA degradation during MHV68 infection. The majority of viral mRNAs are targeted by muSOX during a lytic infection, whereas escapees are enriched for viral noncoding RNAs. The decreased viral mRNA levels in a wild-type MHV68 infection dampens viral protein accumulation and directly influences the composition of progeny viral particles. This, in turn, impacts early events in subsequent rounds of infection prior to the onset of host shutoff. Finally, we demonstrate that inhibiting this global virus and host mRNA degradation restricts MHV68 replication in a cell type specific manner both in cell culture and *in vivo*. Our findings reveal a new layer of complexity underlying gammaherpesvirus gene expression control, and highlight the importance of mRNA degradation in the viral life cycle.

## Results

### Expression of the majority of viral mRNAs is dampened during host shutoff

**Figure 1:**
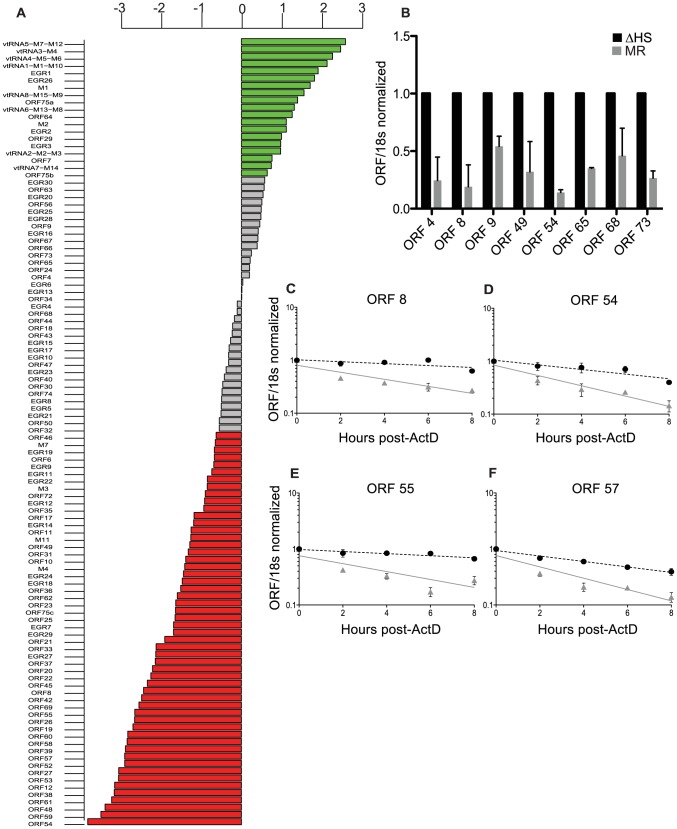
Expression of the majority of viral mRNAs is dampened during host shutoff. (A) For viral genes, the log_2_ fold change in expression upon infection with WT compared to ΔHS MHV68 expression was plotted and data points were colored to indicate the adjusted P values, with green points indicating positive log_2_ fold change with P value<0.05 and red indicating negative log_2_ fold change with P value<0.05. (B) RT-qPCR was used to validate selective viral transcripts. RNA was isolated at 24 hpi from NIH 3T3 cells infected with MR or ΔHS MHV68 at an MOI of 5. Transcript levels were normalized to 18 s and ΔHS levels set to 1. (C–F) mRNA half-life analyses were conducted by infecting NIH 3T3 cells with MR or ΔHS MHV68 at an MOI of 5. At 18 hpi, 2 ug of Actinomycin D was added to block transcription and RNA was harvested at the indicated times thereafter. RT-qPCR was performed with ORF-specific primers and probes to determine mRNA levels. The black dotted line indicates the best-fit line for the ΔHS virus, and the grey solid line indicates the best-fit line for the MR virus.

The gammaherpesviral SOX protein drives widespread degradation of cellular mRNAs during lytic infection, yet the extent to which viral transcripts escape this fate remains unknown. We addressed this question by comparing an MHV68 mutant that is impaired for host shutoff (ΔHS; host shutoff defective) with its matched mutant rescue (MR) virus in which the mutation was restored to wild type. ΔHS contains an R443I mutation located in the ORF37 gene encoding the SOX homolog (MHV68 muSOX) that renders muSOX selectively defective for mRNA degradation [1]. Viral transcript abundance was comprehensively evaluated using an MHV68 microarray platform containing 12,000 tiled 60-mer probes that provides 3-fold coverage of each strand of the viral genome. Relative transcript levels were measured in NIH 3T3 cells infected at an MOI of 5 at 24 hours post infection (hpi), a time at which the mRNA degradation phenotype is well established. Surprisingly, the majority of viral mRNAs from all three kinetic classes (immediate early (IE), early (E), and late (L)) were significantly downregulated during a MR infection as compared to a ΔHS infection, suggesting that viral transcripts do not broadly escape muSOX-induced degradation (Figure 1A, red bars). This trend was confirmed using RT-qPCR as an independent measure of viral mRNA levels during infection for three representative genes (ORFs 8, 49, and 54) after normalization to the host shutoff resistant 18S ribosomal RNA [16], [20]. Furthermore, RT-qPCR analysis of several genes that appeared unchanged in the array data (ORFs 4, 9, 65, 68, and 73) indicated that these were also decreased during infection with MR relative to ΔHS MHV68, suggesting that the microarray results may be an underestimation of the extent of viral mRNA targeting by muSOX (Figure 1B). RT-qPCR analysis of these transcripts in both a murine dendritic cell line (DC2.4) and in primary murine embryonic fibroblasts (MEF) infected with the MR or ΔHS virus yielded similar results, indicating that muSOX targeting of viral transcripts during infection occurs in multiple cell types (Figure S2A–B).

The reduced viral transcript levels could either be due to cleavage of viral mRNAs by muSOX, or be a secondary consequence of altered levels of cellular proteins (e.g. transcription factors) affected by host shutoff. To distinguish these possibilities, we compared the half-life of representative IE (ORF57), E (ORFs 54 and 55), and L (ORF8) viral transcripts following infection of 3T3 cells with MR or ΔHS virus. The decay rate of each mRNA was calculated following addition of actinomycin D (ActD) to halt transcription at 18 hpi. In all cases, the ΔHS mutation led to a significant increase in transcript stability, indicating they are directly targeted for degradation by muSOX (Figure 1C–F). Thus, host shutoff is not restricted to cellular mRNAs, but also broadly impacts viral mRNA abundance during gammaherpesvirus infection.

### Noncoding RNAs are enriched in the escapee population

**Figure 2:**
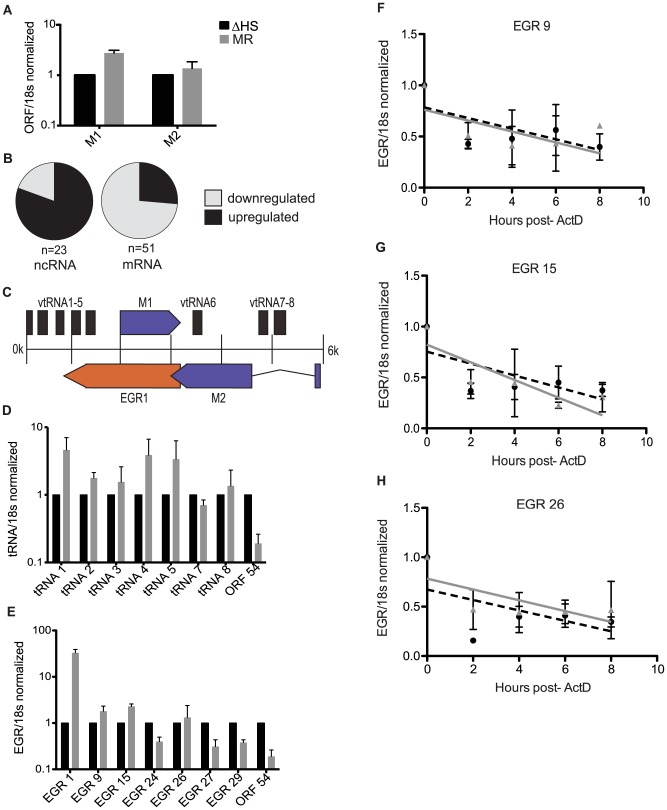
Noncoding RNAs are enriched in the escapee population. (A) RT-qPCR on viral transcripts M1 and M2. RNA was harvested from infected NIH 3T3 cells at 24 hpi. Viral transcripts were normalized to 18 s and ΔHS levels set to 1. (B) The array data was used to determine what percentage of viral noncoding RNAs (ncRNAs) are upregulated during a MR infection (70%), and what percentage of viral mRNAs are downregulated (83.3%). (C) Schematic of the left end of the MHV68 genome, including the 8 viral tRNAs, Expressed Genomic Region (EGR 1), and ORFs M1 and M2. (D and E) RT-qPCR on viral ncRNA was done by harvesting RNA from infected NIH 3T3 cells at 24 hpi. cDNA was made using transcript specific reverse primers and each transcript was normalized to 18 s and ΔHS levels set to 1. ORF54 was used as a control to show downregulation. 3–5 independent RT-qPCRs were done for each transcript. (F–H) EGR half-life analyses were conducted by infecting NIH 3T3 cells with MR or ΔHS MHV68 at an MOI of 5. At 18 hpi, 2 ug of Actinomycin D was added to block transcription and RNA was harvested at the indicated times thereafter. RT-qPCR was performed with EGR-specific primers to determine transcript levels. The black dotted line indicates the best-fit line for the ΔHS virus, and the grey solid line indicates the best-fit line for the MR virus.

Although the majority of MHV68 transcripts appear subject to degradation during host shutoff, a subset may escape this fate (Figure 1A, green bars). For example, levels of the M1 and M2 mRNAs are higher in MR infected cells relative to ΔHS infection as measured both by microarray analysis and RT-qPCR (Figure 2A). Interestingly, many of the other putative escapees are candidate or confirmed non-coding RNAs (ncRNAs). Indeed, analysis of the viral mRNA and ncRNA distribution revealed that most of the viral mRNAs fall under the muSOX-susceptible category, whereas the population of muSOX escapees is strongly enriched for ncRNAs (Figure 2B). This observation correlates well with previous data indicating that the gammaherpesviral SOX proteins preferentially target translationally competent cellular mRNAs.

To confirm that noncoding transcripts are not depleted by muSOX, we compared the abundance of seven of the RNA polymerase III (Pol III) transcribed viral tRNAs during infection with MR or ΔHS virus at 24 hpi by RT-qPCR (Figure 2C–D). In contrast to the viral ORF54 mRNA, none of the tRNA levels were decreased during a MR infection relative to infection with the ΔHS virus. We next examined several of the Expressed Genomic Regions (EGRs), which are RNA polymerase II (Pol II) transcripts distributed throughout the MHV68 genome that lack apparent coding capacity [23]. Similar to the viral tRNAs and in accordance with the microarray data, qPCR analysis indicated that several of the EGRs escape downregulation by muSOX, a finding confirmed by half-life analysis for EGRs 9, 15, and 26 (Figure 1A, 2E–H). Some EGRs, such as EGR1, were modestly or markedly upregulated during a MR infection, though this was due to a secondary transcriptional increase rather than enhanced RNA stability (data not shown). We noted that not all of the EGRs escape the effects of host shutoff, as EGRs 24, 27, and 29 were decreased during a MR relative to ΔHS MHV68 infection (Figure 2E). Given that the EGRs have yet to be functionally characterized, it is possible that those that are decreased in the presence of active muSOX are not truly noncoding, or may possess certain mRNA-like features. Collectively, however, these data suggest that muSOX preferentially targets viral mRNAs for degradation, while many viral ncRNAs are unaffected (or upregulated) during host shutoff.

### RNA degradation alters intracellular viral protein levels and virion composition

**Figure 3:**
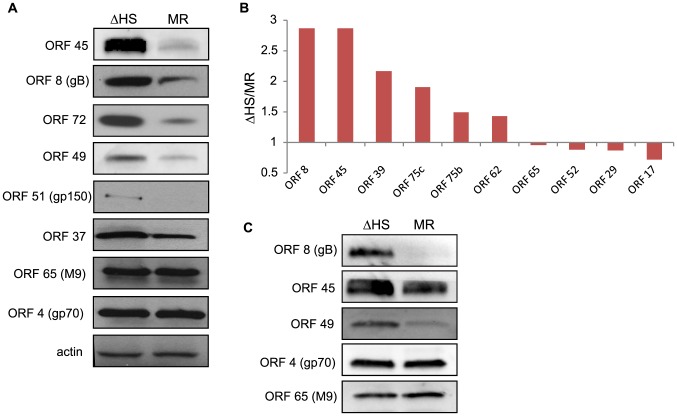
RNA degradation alters intracellular viral protein levels and virion composition. (A) To compare the accumulation of viral proteins during infection with ΔHS and MR MHV68, NIH 3T3 cells were infected at MOI of 5 and cell lysates collected at 24 hpi. Viral proteins were detected using antibodies against ORF 45, 8, 72, 49, 51, 37, 65, and 4. Actin was used as a loading control. (B) Relative abundance of some virion proteins comparing ΔHS levels over MR based on mass spectrometry (MS) peptide counts. Virions were isolated by sucrose gradient centrifugation and run through MS. Peptide numbers were normalized to major capsid ORF 25 and genome number as determined by qPCR. Graph includes data from two independent MS runs. (C) Virions were isolated by sucrose gradient centrifugation and abundance of the virion proteins ORF 8, 45, 49, 4, and 65 were determined by Western blot.

We reasoned that if muSOX-induced depletion of viral mRNAs was important for regulating viral gene expression, then the differences in mRNA levels should lead to corresponding alterations in viral protein abundance. Protein levels were therefore measured for viral factors with available antibodies by Western blotting lysates of NIH 3T3 cells infected with MR or ΔHS MHV68. Indeed, in accordance with the mRNA abundance data, ΔHS infected cells contained higher protein levels of ORF51 (gp150), ORF8 (gB), ORF45, ORF37 (muSOX), ORF49, and ORF72 (v-Cyclin) relative to cells infected with MR virus (Figure 3A). Not all of the mRNA abundance changes resulted in altered protein levels however, as ORF4 (gp70) and ORF65 (M9) proteins accumulated to similar levels during MR and ΔHS infections even though their transcripts were targeted by muSOX. These results indicate that in most cases, muSOX targeting of viral mRNAs regulates protein abundance during MR infection.

How might the broad muSOX-driven reduction in viral gene expression impact the outcome of infection? One possibility is that increased abundance of select viral proteins in the absence of functional muSOX might lead to alterations in viral particle composition, for example through elevated concentrations of envelope or tegument proteins. In particular, it has been shown that tegument composition can be influenced by intracellular protein concentration [24]. We therefore analyzed the composition of virions purified from MR or ΔHS infected cells by mass spectrometry (MS). Figure 3B shows the relative abundance of proteins detected in both MR and ΔHS virions from two MS runs (several proteins were detected in only 1 sample and thus were excluded from the comparative analysis; a full list of MS results are provided in Table S1). To ensure that differences were not due to unequal viral particle numbers between samples, peptide counts were first normalized to the major capsid protein ORF25, as capsid composition is geometrically constrained and thus should not be influenced by intracellular protein abundance. We then performed a secondary normalization against genome number (Table 1). Among the viral proteins available for comparison, there was a 2-3-fold increase in the abundance of select tegument (ORFs 45, 49, and 75c) and envelope (ORF8; gB) proteins in ΔHS virions relative to MR virions (Figure 3B). As expected, the levels of the minor capsid protein ORF65 and minor scaffolding protein ORF17 remained unchanged, validating the normalization strategy. Tegument proteins ORF75b and ORF52 appeared unchanged as well, indicating that not all components of the virion were altered. To confirm these data and compare the abundance of additional virion components not detected in the MS, purified viral particles were resolved by SDS-PAGE and Western blotted with antibodies against ORFs 4, 8, 45, 49, and 65. Indeed, tegument proteins ORF45 and ORF49 and the glycoprotein ORF8 (gB) accumulated to higher levels in ΔHS virions (Figure 3C). In contrast, we observed no differences in the relative abundance of the ORF65 minor capsid protein or ORF4 (gp70). To confirm that altered virion composition in the absence of host shutoff was not restricted to virions derived from 3T3 cells, we compared the levels of the muSOX-impacted tegument proteins ORF45 and ORF49 by Western blot of virions purified from infected MEFs, and observed a similar increase in their levels in the ΔHS virus (Figure S3). Thus, in the absence of muSOX-induced mRNA degradation, the abundance of several viral proteins increases, resulting in altered composition of progeny virions.

**Table 1:** Normalization strategy for mass spectrometry results.

	MR			ΔHS		
			Genome #			Genome #
ORF		ORF/capsid	a42100000		ORF/capsid	a21100000
capsid 25	1.22E-06	-	-	6.31E-07	-	-
ORF75c	7.52E-07	0.618955524	1.47E-08	3.72E-07	0.589510263	2.79E-08
ORF75b	5.57E-07	0.457868722	1.09E-08	2.17E-07	0.343556856	1.63E-08
gB	1.85E-07	0.151811516	3.61E-09	1.38E-07	0.218056541	1.03E-08
gM	2.96E-07	0.243808397	5.79E-09	1.68E-07	0.265301148	1.26E-08
ORF29	2.25E-07	0.185355195	4.40E-09	5.09E-08	0.080678019	3.82E-09
ORF59	8.43E-08	0.069367842	1.65E-09	1.40E-07	0.221416547	1.05E-08
			b9220000			b5880000
capsid 25	1.57E-06			1.33E-06		
ORF62	7.81E-07	0.496152539	5.38E-08	6.02E-07	0.45300884	7.70E-08
ORF52	2.51E-06	1.595994443	1.73E-07	1.19E-06	0.896746041	1.53E-07
ORF65	1.30E-06	0.823241982	8.93E-08	6.65E-07	0.501103815	8.52E-08
ORF59	2.05E-07	0.130477608	1.42E-08	7.38E-07	0.555948069	9.45E-08
ORF17	3.73E-07	0.236822723	2.57E-08	1.44E-07	0.108114721	1.84E-08
ORF45	2.56E-07	0.162445417	1.76E-08	3.94E-07	0.296639457	5.04E-08

Top portion of the table are proteins identified in both ΔHS and MR samples for the first mass spectrometry (MS) run. Lower portion of the table is from the second MS run. The peptide count was normalized to major capsid protein ORF 25, and subsequently normalized to genome number, as determined by qPCR.

^a^ Genome number for first MS run.

^b^ Genome number for second MS run.

### Altered virion composition leads to enhanced cell surface binding and entry

**Figure 4:**
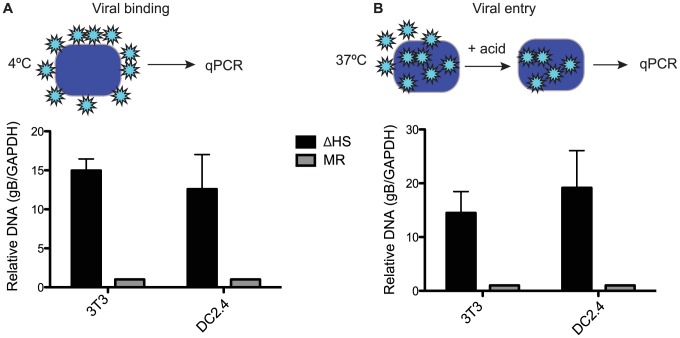
Altered virion composition leads to enhanced cell surface binding and entry. (A) Schematic showing cells were infected at an MOI of 5 at 4°C to allow viral binding, but prevent uptake. Both NIH 3T3 cells and DC2.4 cells were infected with MR or ΔHS MHV68. Cells were washed 4X with PBS at 90 min post infection, DNA was isolated, and qPCR used to quantify relative DNA levels by normalizing gB to GAPDH levels and setting MR levels to 1. (B) Cells were infected at an MOI of 5 at 37°C to allow uptake of virions. At 90 min post infection, the viral particles not internalized were stripped from the surface by the addition of 40 mM citric acid for 5 minutes and washed 4X with PBS. DNA was isolated and qPCR used to quantify relative DNA levels of internalized virus. Each graph represents 3 independent experiments.

We next sought to determine whether the differences in MR and ΔHS MHV68 particles resulted in any functional distinctions during a *de novo* infection. Given that viral envelope glycoproteins are involved in cell surface binding and internalization, we hypothesized that increased glycoprotein concentrations such as those we observed for gB might influence these events. We first measured viral attachment to NIH 3T3 cells and to the dendritic cell line DC2.4 by incubating them with MR or ΔHS MHV68 for 90 min at 4°C to allow attachment but prevent uptake, then measuring the relative level of attached virions by qPCR for the viral genome (Figure 4A). Indeed, there was a marked (10–15 fold) increase in ΔHS virus binding relative to MR binding in both cells types (Figure 4A). We observed similar results when we quantified viral entry by incubating at 37°C for 90 min, acid stripping, and measuring intracellular viral particles by qPCR, indicating that the bound particles were successfully internalized (Figure 4B). This increase in binding and entry of ΔHS virions was not a secondary consequence of excess defective particles, as we observed no significant differences in the particle∶PFU ratio between the WT, MR and ΔHS viruses (Figure S4). We conclude that the altered virion composition of ΔHS directly influences the first step of the viral lifecycle.

### Failure to degrade viral mRNAs leads to enhanced lytic cycle entry

**Figure 5:**
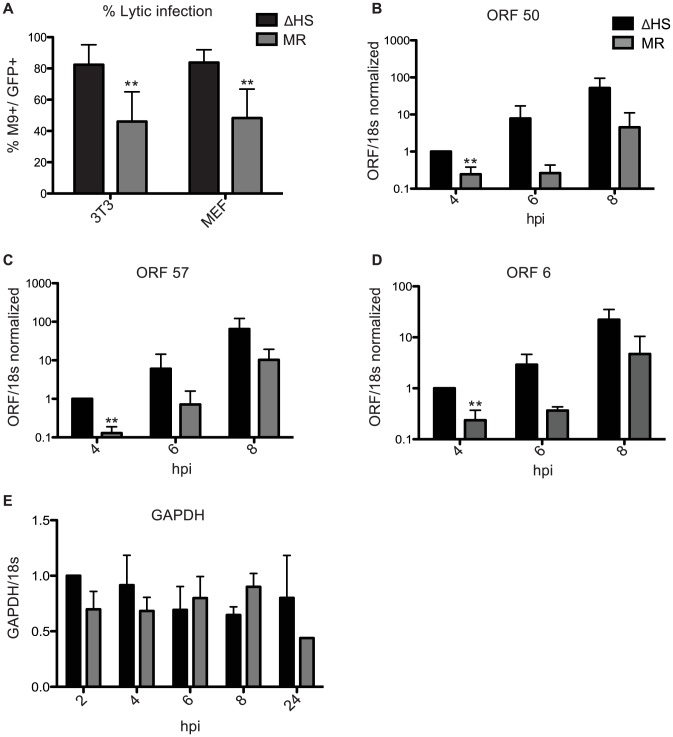
Failure to degrade viral mRNAs leads to enhanced lytic cycle entry. (A) Shown is the percent of lytic-expressing infected NIH 3T3 or MEF cells. Cells were infected at an MOI of 5 with GFP-BAC MHV68 MR or ΔHS and were analyzed at 18 hpi for GFP and M9 expression by immunofluorescence using anti-M9 antibodies. 5 fields of view from three independent experiments were counted and the percentage of GFP+M9+ cells calculated. ** Indicates p-value<0.01, determined by student t-test. (B–D) To measure levels of RTA-responsive transcripts after infection with MR or ΔHS MHV68, NIH 3T3 cells were infected at an MOI of 5 and RNA harvested at indicated times post infection. RT-qPCR was used to quantify relative levels of ORF 50 (B), ORF 57 (C), and ORF 6 (D). (E) GAPDH levels were measured to show that host shutoff had not yet initiated at the 8 hpi time point.

We next examined whether the altered virion composition influenced progression of the MHV68 lifecyle post entry. In particular, tegument proteins are deposited directly into newly infected cells and thus are poised to have an early impact on the course of infection. A key early outcome of a herpesvirus infection is whether the incoming virus establishes latency or progresses to lytic replication, with latency generally favored upon gammaherpesvirus infection *in vivo* and in cultured cells. Although MHV68 is atypical in that in cell culture (unlike *in vivo*) it defaults to lytic replication, we previously observed that not all infected cells immediately enter the lytic cycle and a larger proportion of NIH 3T3 cells displayed a lytic marker (muSOX) upon infection with the ΔHS virus compared to the MR virus [1]. To determine whether the enhanced lytic entry phenotype of ΔHS MHV68 was specific to NIH3T3 cells or represented a more general effect, we measured the proportion of infected cells expressing the lytic protein ORF65 (M9) in both NIH 3T3 cells and mouse embryo fibroblasts (MEFs) at 18 hpi (Figure 5A). M9 was used as a lytic marker because its levels are not influenced by muSOX activity (see Figure 3A). Both viruses constitutively express GFP, which serves as a marker of infected cells. Indeed, in both NIH 3T3 and MEF cells 82.4% and 84.3%, respectively, of the GFP+ infected cells were also M9+ during infection with ΔHS virus. However, only 46.6% and 50.9% of NIH 3T3 and MEF cells infected with the MR virus were double positive (Figure 5A). Thus, RNA degradation by muSOX tempers viral lytic cycle entry during a *de novo* infection.

We hypothesized that the increased number of ΔHS infected cells directly entering the lytic cycle might be a consequence of enhanced activity of RTA, the major lytic transactivator. Although RTA is not a component of MHV68 particles, it is an IE gene whose activity is influenced by tegument proteins [25], [26], some of which are over represented in ΔHS virions (Figure 3B–C). We assessed RTA activity at early times post infection by measuring levels of the RTA-responsive transcripts ORF 50 (RTA), ORF 57, and ORF 6 by RT-qPCR (Figure 5B–D). All three RTA-responsive transcripts were induced to significantly higher levels at 4 hpi (p-value<0.007) after infection with the ΔHS virus as compared to the MR virus. A similar trend was observed at 6 and 8 hpi, although the differences at these time points were not statistically significant. Importantly, the levels of the cellular GAPDH mRNA remained unchanged between 2–8 hpi but, as expected, decreased at 24 hpi during host shutoff (Figure 5E), which generally starts 10–18 hpi [15]. This confirmed that the effects on viral transcript accumulation manifested prior to the onset of muSOX-induced mRNA degradation, and therefore must derive from differences in the incoming viral particles. Thus, RTA activity is stimulated shortly after infection with the ΔHS virus, likely due to higher levels of RTA activators within the virion itself.

### muSOX-induced mRNA degradation is important for viral amplification in a cell type specific manner *in vitro* and *in vivo*

**Figure 6:**
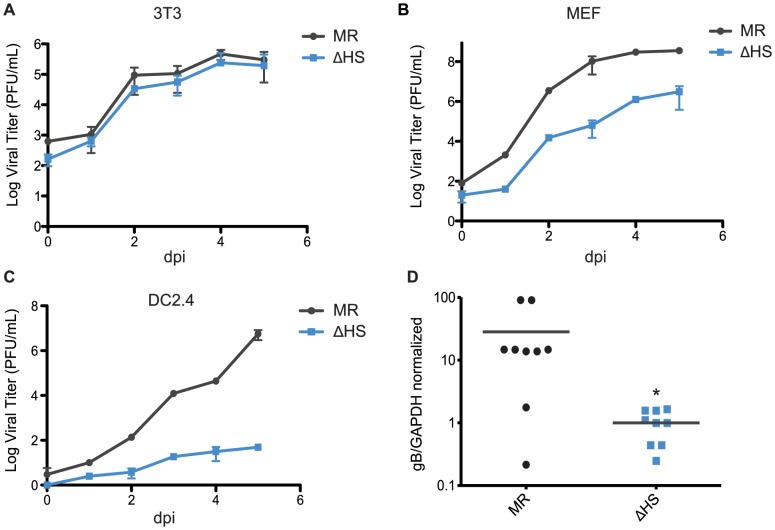
muSOX-induced mRNA degradation is important for viral amplification in a cell type specific manner in vitro and in vivo. (A–C) Multi-step growth curves were done in (A) NIH 3T3 cells, (B) murine embryonic fibroblasts, or (C) DC2.4 cells. Cells were infected at an MOI of 0.05 with MR or ΔHS MHV68, cells and supernatant collected at the indicated times post infection, and the titer was determined by plaque assay. At least three independent experiments were performed for each cell type. (D) C57BL/6 mice were infected by the intraperitoneal route with 1×10^3^ pfu of MR or ΔHS MHV68. At 10 dpi spleens were harvested, homogenized, DNA extracted, and qPCR used to quantify viral particles. Each dot represents the relative value from a single spleen, and the bar indicates the mean value for each virus. * Indicates p-value<0.05.

Given the enhanced viral gene expression and lytic cycle entry phenotypes of the ΔHS virus, it might be predicted that this mutant virus would replicate more efficiently, as has been observed for MHV68 engineered to constitutively express RTA [27], [28]. However, ΔHS was shown to have no growth advantage over MR in NIH 3T3 cells and, furthermore, in mice it displayed defects in trafficking from the lung to distal sites, as well as severely decreased abundance in splenocytes at 2 weeks post infection during peak latency establishment [1]. These observations suggest that there may be cell type specific replication defects associated with impaired muSOX RNA degradation activity that manifest subsequent to the viral entry and gene expression enhancements observed above. We therefore compared the replication rates of the MR and ΔHS viruses in NIH 3T3, MEF, and DC2.4 cells using multi-step growth curves. As reported previously, the MR and ΔHS viruses grew to similar titers in NIH 3T3 cells (Figure 6A). However, ΔHS displayed moderately delayed kinetics in MEF cells, resulting in a 2 log defect at 5 dpi (Figure 6B) and severely delayed kinetics in DC2.4 cells, resulting in a 5 log defect at 5 dpi (Figure 6C). The DC2.4 result is notable given that MHV68 infects and traffics through dendritic cells in mice [29]. Thus, although the ΔHS virus binds and enters these cells more efficiently than MR, its failure to control gene expression through muSOX activity results in a downstream amplification defect.

B cells are key sites of viral replication and latency establishment *in vivo*, however these cells cannot be readily infected in culture. We therefore sought to monitor viral replication in these cells in infected mice. MHV68 infection via the intraperitoneal route (i.p.) leads to lytic replication in macrophages and B cells in the spleen that peaks at 10 dpi, whereupon the lytically infected cells are largely cleared prior to the onset of peak latency at 18 dpi [30], [31]. We therefore measured viral load in splenocytes of mice infected i.p. with each virus at 10 dpi during the lytic replication phase, and found significantly reduced viral titers with ΔHS compared to MR (Figure 6D). These data indicate that inhibition of muSOX-induced RNA degradation restricts MHV68 amplification in a cell type specific manner both *in vitro* and *in vivo*.

## Discussion

Our findings demonstrate that the majority of gammaherpesviral mRNAs are targeted by the muSOX nuclease and thus reveal a novel layer of regulation in viral gene expression. The importance of this activity is highlighted by the alterations in virion composition, as well as in viral entry, early lytic gene induction, and viral amplification that manifest in the absence of functional muSOX. Thus, controlling viral mRNA abundance through enhanced degradation is a regulatory activity that exerts influence throughout the viral lifecycle. This gammaherpesviral strategy is distinct from that of all other host shutoff-inducing viruses characterized to date, each of which has evolved mechanisms to selectively target host gene expression. Even when comparing the gammaherpesviruses to the related alphaherpesviruses such as HSV-1, which also induce mRNA cleavage, several distinctions are apparent in the regulation and function of their host shutoff activities. For example, the HSV-1 host shutoff factor vhs is packaged into the viral particle and therefore promotes RNA degradation immediately after viral entry, before the onset of most viral gene expression [2], [10]. It has been proposed that vhs-induced degradation of immediate early HSV-1 mRNAs may facilitate the transition between immediate early and early viral gene expression [32]. However, at least three other viral proteins, VP16, VP22, and UL47, subsequently interact with vhs and restrict its activity against viral transcripts expressed in the early and late kinetic classes [12], [33]–[35]. In contrast, the gammaherpesviral SOX protein is not packaged in virions and is expressed with early kinetics beginning at 8–10 hpi [15], [36]–[39]. Global mRNA degradation occurs following SOX expression and continues for the remainder of the viral lifecycle [14], [15]. Unlike alphaherpesviruses, where there are distinct transitions between each kinetic class of viral genes, during gammaherpesvirus infection the onset of each new phase does not coincide with the downregulation of the prior class of viral genes. Thus, muSOX continues to accumulate even during the final stages of the viral replicative cycle, resulting in degradation of viral transcripts in both early and late kinetic classes.

The targeting of viral and cellular mRNA by muSOX is likely to occur via similar mechanisms. It has been previously shown that SOX and its homologs preferentially cleave RNAs that have been transcribed by Pol II and are translationally competent, but fail to degrade RNAs transcribed by RNA Pol I or III [16], [20]. In agreement with these data, we observed a similar preference for viral mRNAs over ncRNAs in the muSOX targets. In particular, none of the RNA Pol III transcribed viral tRNAs were depleted by muSOX and there was a clear enrichment for other putative ncRNAs (EGRs) in the escapee population. The susceptibility of certain EGRs to muSOX may reflect differences in their composition, such as the presence of small ORFs, or in their localization or potential regulatory activities. Our findings may therefore be useful for future studies aimed at probing the function of the EGRs. Though the majority of viral mRNAs appear targeted by muSOX, there are coding transcripts that escape depletion. This has also been observed for a population of cellular mRNAs [40], [41], which presumably escape either because they lack a SOX-targeting element(s) or because they contain specific protective features, as has been demonstrated recently for the cellular interleukin 6 transcript [42]. The select viral mRNAs refractory to muSOX-induced turnover may exhibit a similar diversity of protective features, and delineation of the precise muSOX-targeting RNA element(s) should shed light on this issue. In addition, whether their selective protection impacts viral replication or pathogenesis remains an important question for future study.

A particularly striking finding was that muSOX-induced degradation of viral mRNAs influenced events that occur during the first 6 hpi, before either its expression or host shutoff initiates. These effects manifested as a consequence of alterations in virion composition imposed during the prior round of replication. There is evidence suggesting that extensive protein-protein interactions dictate tegument composition and deleting certain proteins can alter this balance [24], [43], [44]. Furthermore, pseudorabies virions lacking select tegument proteins accumulate actin in the viral particles, suggesting there is a critical mass that is filled in relation to protein abundance in an infected cell [24], [45]. These findings and others, together with our observations, indicate that tegumentation is a highly orchestrated process regulated through specific protein interactions as well as by protein abundance, which can be at least partially controlled at the level of viral mRNA stability. We anticipate that in addition to the protein composition changes examined herein, there are presumably increased levels of viral and perhaps cellular mRNAs packaged into the ΔHS virions that may also impact early events in the viral lifecycle. Indeed, it has been previously observed that RNAs can be packaged into herpesviral particles in a manner largely linked to their intracellular abundance [46], [47].

In addition to potential structural roles in the virion, tegument proteins can manipulate the cellular environment immediately after their release and thus are thought to play key roles in facilitating infection [48]. For example, immune evasion is partly mediated through the activities of KSHV ORF36 and its homologs, which inhibit the type 1 IFN response [49], as well as HSV-1 UL41, which downregulates MHCII on the cell surface [50]. Other tegument proteins are involved in augmenting early viral gene expression, such as HSV-1 VP16, HCMV pp71, and KSHV RTA, which act as lytic transactivators through coordination with other viral and cellular factors [37], [39], [51], [52]. Unlike in KSHV, RTA is not packaged into the MHV68 virion, although it is expressed with immediate early kinetics and several tegument proteins facilitate lytic infection by boosting its activity. For example, MHV68 ORF49 interacts with PARP-1, a cellular inhibitor of RTA [25], while ORF75c degrades PML-NBs, which interfere with lytic cycle progression [26]. Our observation that cells infected with the ΔHS virus displayed enhanced RTA activity shortly after infection confirms that failure to moderate viral protein levels through mRNA degradation has marked consequences for subsequent rounds of infection, including preferential or enhanced immediate early lytic gene expression.

MHV68 infects a variety of cell types *in vitro* with varying outcomes, including efficient lytic replication in fibroblasts, delayed lytic entry in dendritic cells, and primarily latent or abortive lytic infection in macrophages [53], [54]. *In vivo*, evidence suggests that epithelial cells are lytically infected, while dendritic cells, macrophages, and B cells mainly support latency after an initial burst of lytic infection to amplify the virus and seed a population of infected cells [31], [55]–[57]. Previously, we reported that the MHV68 ΔHS virus exhibits dramatically reduced viral loads at 17 dpi during peak latency [1]. Here, we found that the ΔHS virus also exhibits cell type specific defects during the lytic amplification stage both *in vivo* and in cultured cells. Thus, despite the enhanced lytic gene expression phenotype of the ΔHS virus, one or more subsequent steps in the replication cycle are impaired in certain cell types. Though the basis for the cell autonomous replication defect remains unknown, the reduced amplification *in vivo* could manifest in part through impaired immune evasion, for example as a consequence of elevated viral antigen levels or abortive infection. One possibility is that the innate immune sensing and response mechanisms are differentially tuned in a cell type specific manner, such that failure to degrade viral and host mRNAs triggers a more robust antiviral response in certain cell types. Additionally, we have noted that the WT MHV68 lifecycle progresses more slowly in dendritic cells relative to 3T3 cells or MEFs (unpublished observations), perhaps making these cells more impacted by the increased accumulation of viral mRNAs in the ΔHS infection.

Our data challenge the assumption that viruses that dampen host gene expression do so categorically to provide a competitive advantage to viral gene expression. Instead, we reveal that degradation of viral transcripts during lytic gammaherpesviral infection is integral to establishing the appropriate balance of viral factors necessary for replication and optimal virion composition. Clearly, degradation of viral mRNAs is not absolute and mechanisms may exist to temper the effects of muSOX to ensure adequate accumulation of viral transcripts. For example, it has been shown that KSHV ORF57 can stabilize viral RNA in the nucleus and cytoplasm [58], [59]. Additionally, viral mRNAs may contain regulatory sequences that help modulate their stability. Deciphering how the many layers of gene expression regulation are coordinated during infection remains an important future challenge.

## Materials and Methods

### Ethics statement

This study was carried out in strict accordance with the recommendations in the Guide for the Care and Use of Laboratory Animals of the National Institutes of Health. The protocol was approved by the Committee on the Ethics of Animal Experiments of the University of California Berkeley (approval number R292). All animals were anesthetized prior to infection with isoflurane, and all efforts were made to minimize suffering.

### Cells, viruses, and infections

NIH 3T3, NIH 3T12, Vero, DC2.4, and MEF cells were maintained in Dulbecco's modified Eagle medium (DMEM; Invitrogen) supplemented with 10% fetal bovine serum (FBS; Invitrogen). The green fluorescent protein (GFP)-expressing MHV68 bacterial artificial chromosome (BAC) has been described elsewhere [60], and the R443I host shutoff mutant was previously generated by allelic exchange as previously described [1]. BAC-derived MHV68 virus was produced by transfecting BAC DNA into NIH 3T3 cells using SuperFect (Qiagen). Virus was then amplified in NIH 3T12 cells and titered by plaque assay on NIH 3T3 cells. Before infecting mice, the loxP-flanked BAC vector sequence was removed by passaging the virus through Vero cells expressing Cre recombinase (kindly provided by Dr. Samuel Speck, Emory University).

### Tiled microarray hybridization and analysis

Array data was derived from two independent biological replicates of each infection condition. Custom MHV68 tiled arrays in the 4 by 44,000 format were designed as described previously [36]. 1 µg of RNA was reverse transcribed using an oligo(dT) promoter-primer, subjected to linear amplification and Cy3 or Cy5 labeling using an Agilent Quick Amp labeling kit. Adenovirus spike-in controls were added to each labeling reaction to allow normalization per the two-color spike-in kit instructions (Agilent). The Cy5-labeled reference RNA derived from an independent infection of NIH 3T3 cells at 8 hpi with WT MHV68 was generated, purified, fragmented, and then hybridized in parallel with the Cy3-labeled sample RNA at 65°C for 17 h. Microarrays were scanned with Agilent Scanner Control software (version 7.0), and hybridization signal intensities were quantified using Agilent Feature Extraction software (version 9.5).

Raw data were processed by subtracting the median background signals from the mean signals in the Agilent feature extraction file and then normalized by multiplying the log_2_ values of the probe intensities by the spike-in scale factor for each array. The scaling factor was calculated from the linear fit of the spike-ins versus their concentration. The normalized log_2_ Cy5-labeled reference RNA signal was subtracted from the normalized log_2_ Cy3-labeled experimental sample RNA signal for each probe. The expression value of each viral ORF was calculated as the median of the normalized signal of all tiling probes enclosed inside the ORF. Quality control analyses indicated strong correlation between the biological replicates (Figure S1). Differentially expressed genes were identified after performing empirical Bayes moderated t-statistics using the Bioconductor LIMMA package (version 3.12.1) [61].

### Nucleic acid isolation and measurement

RNA was isolated using the Zymo Mini RNA II Isolation Kit (Zymo Research), treated with Turbo DNase (Ambion) to remove genomic DNA contamination, and reverse transcribed using AMV RT (Promega) with oligo(dT) and 18 s specific primers. For ncRNA analysis, transcript specific reverse primers were used instead of oligo(dT) during cDNA synthesis. cDNA levels were then quantified using DyNAmo color flash SYBR green master mix, ROX passive reference dye (Thermo Scientific), and transcript specific primers (listed in Table S2). Transcript levels were normalized to 18 s. Viral genomes were quantified by isolating DNA using the DNeasy Blood and Tissue kit (Qiagen), and using 10-fold dilutions of 1 ng BAC DNA to generate a standard curve. For mRNA half-life analyses, at 18 hpi 2 µg of actinomycin D (Sigma) was added to halt transcription, and RNA was isolated at the indicated time points for quantification by RT-qPCR using TaqMan reagents (Applied Biosystems). Primers used to detect ORF54 (probe TCACACCTATATCTGTCCAACCCAGCGAA) and ORF55 (probe CCAACCTTTGGCCACGCCCC) are listed in Table S2. Final primer concentrations of 900 nM and probe concentrations of 250 nM were used. ORF57 and ORF8 primers and probes were used as described previously [1], [62]. GAPDH levels were measured using the TaqMan Rodent GAPDH Control and Ribosomal RNA Control reagents (Applied Biosystems). The data were plotted relative to the t = 0 time point which was set to 1.

### Virion isolation and mass spectrometry

Viral supernatants were collected 7 dpi from 6 plates (15 cm) of NIH 3T3 or MEF cells infected at an MOI of 0.5 and centrifuged at 4,000 rpm for 15 min to pellet cellular debris, then subsequently centrifuged over a 20% sucrose cushion at 24,000 rpm for 1 h to pellet the virus. The pellet was resuspended in 200 µl PBS and centrifuged through a 10–60% continuous sucrose gradient at 24,000 rpm for 1 h. Fractions (1 mL) were collected, and DNA was extracted from a portion of each fraction using the DNeasy Blood and Tissue kit (Qiagen) to quantify viral DNA by qPCR using gB-specific primers. Fractions enriched in viral genomes were pooled, pelleted by centrifugation at 24,000 rpm for 1 h, and resuspended in 100 µl of lysis buffer [50 mM Tris-Hcl, pH 7.4, 150 mM NaCl, 2 mM EDTA, 1% Nonidet P-40, 0.1% SDS] for Western blots or 50 µl of ammonium bicarbonate (25 mM) for mass spectrometry.

### Western blots

Cell lysates were prepared in lysis buffer and quantified by Bradford assay. Equivalent amounts of each sample were resolved by SDS-PAGE and Western blotted with the following anti-MHV68 primary antibodies: hybridoma supernatants T1A1 anti-gp150, MG-2C10 anti-gB, and 9c7 anti-gp70, diluted 1∶10 (kindly provided by Phillip Stevenson, University of Cambridge); anti-M9, anti-ORF26, and anti-ORF45, diluted 1∶500 (kindly provided by Ren Sun, UCLA); anti-ORF72, diluted 1∶500 (kindly provided by Linda van Dyk, University of Colorado, Denver); anti-ORF49, diluted 1∶500 (kindly provided by Moon Jung Song, Korea University). Primary antibodies were followed by HRP-conjugated goat anti-mouse or goat anti-rabbit secondary antibodies (Southern Biotechnology, 1∶5000).

### Viral binding and entry assays

NIH 3T3 or DC2.4 cells were infected at 4°C (to measure binding) or 37°C (to measure entry) for 90 min at an MOI of 5. For the binding assay, cells were then washed 4X with ice-cold PBS, scraped, and DNA isolated by DNeasy Blood and Tissue kit (Qiagen). For the entry assay, cells were washed 2X with PBS, and 0.5 ml of citric acid [135 mM NaCl, 10 mM KCl, 40 mM citric acid, pH 3] was added for 5 minutes at RT to strip off remaining cell surface-bound viral particles. Cells were washed twice more with PBS, scraped, and DNA was isolated. Relative genome levels were quantified by qPCR with gB-specific primers.

### Immunofluorescence assays

NIH 3T3 or MEF cells were grown on coverslips and infected with GFP-BAC MHV68 at MOI of 5. At 24 hpi, coverslips were washed with PBS and cells were fixed in 4% formaldehyde for 30 min at RT. Cells were then permeabilized in 1% Triton-X-100 and 0.1% sodium citrate in PBS for 10 min, and incubated with anti-M9 antibodies at 1∶500 in 10% goat serum at 37°C. After 1 hour, coverslips were washed 3X in PBS and incubated with goat anti-rabbit AlexaFluor546 secondary antibody at 1∶1500 (Invitrogen). Coverslips were washed 3X in PBS and mounted in DAPI-containing Vectashield mounting medium (VectorLabs) to stain cell nuclei.

### Growth curves and *in vivo* experiments

For multi-step growth curves, 1.5×10^5^ NIH 3T3, MEF, or DC2.4 cells were infected with MHV68 at MOI of 0.05 and both supernatant and cells were harvested at 0, 1, 2, 3, 4, and 5 dpi and frozen. Samples were freeze-thawed once before titering by plaque assay on NIH 3T3 cells.

For *in vivo* experiments, female C57BL/6J mice were obtained from the Jackson Laboratory (Bar Harbor, ME) and infected when 4–6 weeks old through injection of 1×10^3^ pfu of virus in 0.2 ml PBS into the peritoneal cavity. Spleens were harvested at 10 dpi, and DNA isolated and quantified by qPCR, using gB-specific primers to determine relative levels of viral genomes.

### Supplemental methods

For quality control, the scaled log_2_ Cy3-labeled experimental cRNA probe values and the scaled log_2_ Cy5-labeled reference cRNA probe values for each WT and ΔHS replicate were determined as described above and then compared in box plots using R package ‘graphics’ ignoring outliers [63]. In addition, the scaled log_2_ Cy5-labeled reference cRNA signal was subtracted from the scaled log_2_ Cy3-labeled experimental sample cRNA signal for each probe. The normalized probe values for the WT and ΔHS replicates were analyzed by scatter plot. Linear model fit of the scatter plots was performed using ‘lm’ from R package ‘stats’ [63].

## Supporting Information

Figure S1 **Consistency between array replicates.** (A) Box and whisker plots of the scaled log_2_ Cy3-labeled experimental cRNA probe values (green) and the scaled log_2_ Cy5-labeled reference cRNA probe values (red). (B) Scatter plot and linear model fit (black line) of the normalized probe values of the WT replicates (r^2^ = 0.9622) and DHS replicates (r^2^ = 0.9095).
(PDF)

Figure S2 **Viral transcripts are degraded during MHV68 infection independent of cell type.** DC2.4 (A) or MEF (B) cells were infected with MR or ΔHS MHV68 at MOI of 5 and RNA was harvested 48 hpi (for DC2.4) or 24 hpi (for MEFs). Viral transcript levels were determined by RT-qPCR, and normalized to 18S rRNA. At least 2 independent biological replicates were performed for each cell type.
(EPS)

Figure S3 **Altered virion composition is independent of cell type.** (A) MEFs were infected with MR or ΔHS MHV68 for 5 days, whereupon virions were isolated by sucrose gradient centrifugation and abundance of the virion tegument proteins ORF45 and ORF49, and the capsid protein ORF26 were determined by Western blot. Protein abundance was quantified and graphed relative to the ORF26 capsid protein. At least 2 independent replicates of each Western blot were analyzed.
(EPS)

Figure S4 **The particle to PFU ratio is similar between viruses.** (A) Particle number was determined by isolating DNA from viral stocks and performing qPCR using gB primers. PFU was determined by plaque assay. At least 5 viral stocks from ΔHS and MR, and 3 from WT were assessed for both particle number and PFU. Differences are not statistically significant.
(EPS)

Table S1 **Complete list of proteins identified through mass spectrometry.** All proteins identified by MS, accession number, molecular weight, and peptide count and normalized to protein length and total counts. ^a^First MS run. All proteins listed were identified in either ΔHS or MR samples. ^b^Second MS run. All proteins listed were identified in either ΔHS or MR samples.
(DOCX)

Table S2 **All primers used in this study.** All forward and reverse primers used for each ORF or noncoding RNA.
(DOCX)
