# Supplementary figures and images for: Gammaherpesviral Gene Expression and Virion Composition Are Broadly Controlled by Accelerated mRNA Degradation
Source: PLoS Pathog. 2014 Jan 16;10(1):e1003882. doi: 10.1371/journal.ppat.1003882 (PMC3894220; doi:10.1371/journal.ppat.1003882)

**A**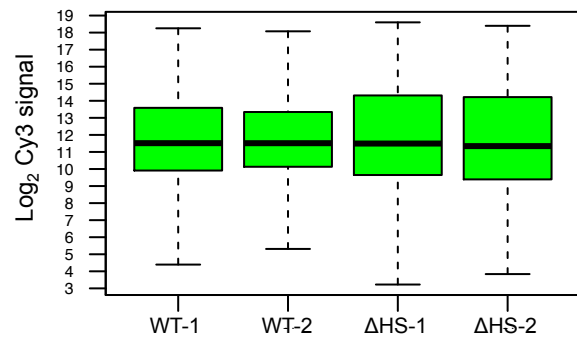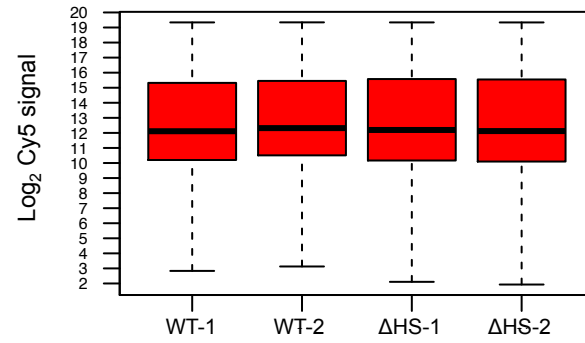**B**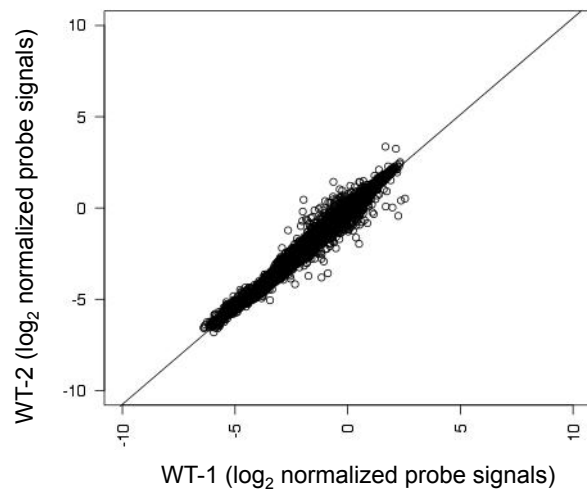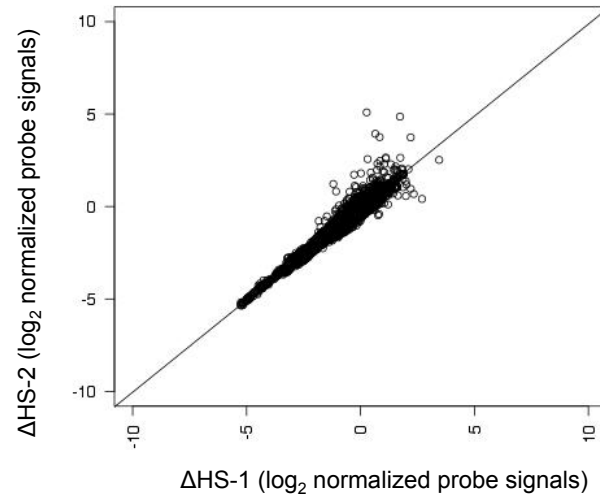

Supplement: Figure S1 — Consistency between array replicates. (A) Box and whisker plots of the scaled log2 Cy3-labeled experimental cRNA probe values (green) and the scaled log2 Cy5-labeled reference cRNA probe values (red). (B) Scatter plot and linear model fit (black line) of the normalized probe values of the WT replicates (r2 = 0.9622) and DHS replicates (r2 = 0.9095). (PDF) [file ppat.1003882.s001.pdf]
