# Supplementary material for: Gammaherpesviral Gene Expression and Virion Composition Are Broadly Controlled by Accelerated mRNA Degradation
Source: PLoS Pathog. 2014 Jan 16;10(1):e1003882. doi: 10.1371/journal.ppat.1003882 (PMC3894220; doi:10.1371/journal.ppat.1003882)
Supplement: Table S1 — Complete list of proteins identified through mass spectrometry. All proteins identified by MS, accession number, molecular weight, and peptide count and normalized to protein length and total counts. aFirst MS run. All proteins listed were identified in either ΔHS or MR samples. bSecond MS run. All proteins listed were identified in either ΔHS or MR samples. (DOCX) [file ppat.1003882.s005.docx]

|  |  |  | MR | | | ΔHS | | |
| --- | --- | --- | --- | --- | --- | --- | --- | --- |
| **ORF** | **Acc #** | **MW** | **Peptide Count** | **Peptide Count normalized by protein lengths** | **Peptide Count normalized by protein lengths & total counts** | **Peptide Count** | **Peptide Count normalized by protein lengths** | **Peptide Count normalized by protein lengths & total counts** |
| ^a^ORF25 | 9629568 | 153276.9 | 158 | 0.0010308 | 1.22E-06 | 66 | 0.00043059 | 6.31E-07 |
| ORF75c | 9629630 | 145761.6 | 93 | 0.000638 | 7.52E-07 | 37 | 0.00025384 | 3.72E-07 |
| ORF75b | 9629631 | 141955.9 | 67 | 0.000472 | 5.57E-07 | 21 | 0.00014793 | 2.17E-07 |
| ORF8 (gB) | 209976837 | 95853.1 | 15 | 0.0001565 | 1.85E-07 | 9 | 9.39E-05 | 1.38E-07 |
| gM | 9629609 | 43768.7 | 11 | 0.0002513 | 2.96E-07 | 5 | 0.00011424 | 1.68E-07 |
| ORF29 | 9629603 | 57571.5 | 11 | 0.0001911 | 2.25E-07 | 2 | 3.47E-05 | 5.09E-08 |
| gH | 9629567 | 82892.6 | 3 | 3.62E-05 | 4.27E-08 |  |  |  |
| ORF59 | 9629621 | 41954.9 | 3 | 7.15E-05 | 8.43E-08 | 4 | 9.53E-05 | 1.40E-07 |
| ORF33 | 9629574 | 35698 | 4 | 0.0001121 | 1.32E-07 |  |  |  |
| ORF21 | 1246776 | 72256.6 | 7 | 9.69E-05 | 1.14E-07 |  |  |  |
| ORF11 | 257782162 | 42550.6 | 2 | 4.70E-05 | 5.54E-08 |  |  |  |
| ORF6 | 13249148 | 123274.1 | 1 | 8.11E-06 | 9.57E-09 |  |  |  |
| ^b^ORF25 | 9629568 | 153276.9 | 84 | 0.000548 | 1.57E-06 | 46 | 0.00030011 | 1.33E-06 |
| ORF62 | 209976844 | 36777.5 | 10 | 0.0002719 | 7.81E-07 | 5 | 0.00013595 | 6.02E-07 |
| ORF75b | 9629631 | 141955.9 | 10 | 7.04E-05 | 2.02E-07 |  |  |  |
| ORF52 | 9629617 | 14863.1 | 13 | 0.0008746 | 2.51E-06 | 4 | 0.00026912 | 1.19E-06 |
| ORF21 | 1246776 | 72256.6 | 11 | 0.0001522 | 4.37E-07 |  |  |  |
| ORF65 | 9629625 | 19948.6 | 9 | 0.0004512 | 1.30E-06 | 3 | 0.00015039 | 6.65E-07 |
| ORF59 | 9629621 | 41954.9 | 3 | 7.15E-05 | 2.05E-07 | 7 | 0.00016685 | 7.38E-07 |
| ORF17 | 209976839 | 30820.1 | 4 | 0.0001298 | 3.73E-07 | 1 | 3.24E-05 | 1.44E-07 |
| ORF48 | 9629570 | 28982.6 | 2 | 6.90E-05 | 1.98E-07 |  |  |  |
| gM | 9629609 | 43768.7 | 3 | 6.85E-05 | 1.97E-07 | 2 | 8.90E-05 | 3.94E-07 |
| ORF45 | 9629612 | 22465.7 | 2 | 8.90E-05 | 2.56E-07 |  |  |  |
| ORF33 | 9629574 | 35698 | 1 | 2.80E-05 | 8.05E-08 |  |  |  |
