# Supplementary material for: Gammaherpesviral Gene Expression and Virion Composition Are Broadly Controlled by Accelerated mRNA Degradation
Source: PLoS Pathog. 2014 Jan 16;10(1):e1003882. doi: 10.1371/journal.ppat.1003882 (PMC3894220; doi:10.1371/journal.ppat.1003882)
Supplement: Table S2 — All primers used in this study. All forward and reverse primers used for each ORF or noncoding RNA. (DOCX) [file ppat.1003882.s006.docx]

| **Transcript** | **Forward** | **Reverse** |
| --- | --- | --- |
| M1 | AAAGCATAGCTCACTGGCCATC | TCCAGCTTCTCGAAGGAATCAG |
| M2 | TAAGGACCTCGTAGAGATTGGC | ACGTTAAAGTCCCCATGGAAGC |
| ORF 4 | GGGATTGTGGGTGTAAATGGTGAC | CAGGGAGTCACGGTTGTCCA |
| ORF 6 | AGGGACAGATTTCCTCAGGTGC | CTGGCGTGGAAGCTGTTACC |
| ORF 8 (gB) | GGCCCAAATTCAATTTGCCT | CCCTGGACAACTCCTCAAGC |
| ORF 9 | CAATTGCTGTATCCCATCTGCG | GGAAACCCACATTCACCCAAAC |
| ORF 49 | TCAGACACTGCGCCACAACT | CTCTCCAATAGTGTTAGGTGTCTGATAATTA |
| ORF 50 | GGCCGCAGACATTTAATGAC | GCCTCAACTTCTCTGGATATGCC |
| ORF 54 | CTCGACACACCTTCCAGTTTGA | GCCAGACCTGCCAAGTACCA |
| ORF 55 | CCACTGACATTGCTGGTATTGTATC | CTATTTGGTCGGGTGACTCACTT |
| ORF 57 | GCTAGACGAACAGTTGGCCTG | TTGGTGCGGTGTGTTTGTCC |
| ORF 65 (M9) | AGACAGGGTCCATCATTTTGGC | TTGGCAAAGACCCAGAAGAAGC |
| ORF 68 | CTCAAATACACTGGCCGCCATC | CGTGCTTGAGATATGAGTGAGT |
| ORF 73 | AAGGGTTGTCTTGGCCTAC TGTG | AGAGATGCTGTGGGACCATGTTG |
| vtRNA 1 | TAGAGCAACAGGTCACCGATC | TGGACCCACTTCCTCGACCAG |
| vtRNA 2 | GGTAGAGCAGCGGTTCCT | ACTCCCCCTCTCAACCA |
| vtRNA 3 | TGGCAGGCCAACATA | CGTGCTCCTCGATGGTCA |
| vtRNA 4 | GCGGCAGGCTCATC | ATCTCAACTCTGCGTCGG |
| vtRNA 5 | CTCCACCTTTAACCAG | TAGAGCATCAGGCTAGTA |
| vtRNA 7 | GAGCGGCAGACACCA | TAGCTGGCCAGGACT |
| vtRNA 8 | \|  \|  \| \| --- \| --- \| \|  \| 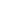 \|  \| CCCATCCTGTTGGTT \| \| --- \| | CGCGGGTAGCTAGTC |
| EGR 1 | TCGATCTGGATCTCACCTTTGCTG | CTTGGATTCTTGGTGGCACCC |
| EGR 9 | ATGACGACCCGGATATTGTAGCAG | GAATGTGAGGAGGAGGACCAAGTC |
| EGR 15 | GGAGATTGGGTCACTAGACAGCAG | CTACCAACCCTGTTTGCCTCCTA |
| EGR 24 | ACTACACAAGGCACAGACCTGG | GTTGAATCTCCACCGTGCTCTGA |
| EGR 26 | CCGCTGGGCATGTTCTACTTTG | GGACAGTATGGTGAATTGATGGGT |
| EGR 27 | ATGTACGTCCAGACCCGTCC | GATTTGTTGAGGTTTGGGTCGGTG |
| EGR 29 | GGTTTGGCGTTTGAGTTCCCTG | CAGGGTGCAAACGTAGGTGC |
| GAPDH | CGGAGTCAACGGATTTGGTCGTAT | AGCCTTCTCCATGGTGGTGAAGAC |
| 18s | GTAACCCGTTGAACCCCATT | CCATCCAATCGGTAGTAGCG |
